# Use of the *piggyBac *transposon to create HIV-1 *gag *transgenic insect cell lines for continuous VLP production

**DOI:** 10.1186/1472-6750-10-30

**Published:** 2010-03-31

**Authors:** Alisson G Lynch, Fiona Tanzer, Malcolm J Fraser, Enid G Shephard, Anna-Lise Williamson, Edward P Rybicki

**Affiliations:** 1 Department of Molecular and Cell Biology, Faculty of Science, University of Cape Town, Rondebosch, Cape Town, South Africa; 2 Institute of Infectious Disease and Molecular Medicine, Faculty of Health Sciences, University of Cape Town, Observatory, Cape Town, South Africa; 3 MRC/UCT Liver Research Centre, Department of Medicine, Faculty of Health Sciences, University of Cape Town, Observatory, Cape Town, South Africa; 4 National Health Laboratory Service, Groote Schuur Hospital, Observatory, Cape Town, South Africa; 5 Department of Biological Sciences, University of Notre Dame, Notre Dame, IN46556-0369, USA

## Abstract

**Background:**

Insect baculovirus-produced Human immunodeficiency virus type 1 (HIV-1) Gag virus-like-particles (VLPs) stimulate good humoral and cell-mediated immune responses in animals and are thought to be suitable as a vaccine candidate. Drawbacks to this production system include contamination of VLP preparations with baculovirus and the necessity for routine maintenance of infectious baculovirus stock. We used *piggyBac *transposition as a novel method to create transgenic insect cell lines for continuous VLP production as an alternative to the baculovirus system.

**Results:**

Transgenic cell lines maintained stable *gag *transgene integration and expression up to 100 cell passages, and although the level of VLPs produced was low compared to baculovirus-produced VLPs, they appeared similar in size and morphology to baculovirus-expressed VLPs. In a murine immunogenicity study, whereas baculovirus-produced VLPs elicited good CD4 immune responses in mice when used to boost a prime with a DNA vaccine, no boost response was elicited by transgenically produced VLPs.

**Conclusion:**

Transgenic insect cells are stable and can produce HIV Pr55 Gag VLPs for over 100 passages: this novel result may simplify strategies aimed at making protein subunit vaccines for HIV. Immunogenicity of the Gag VLPs in mice was less than that of baculovirus-produced VLPs, which may be due to lack of baculovirus glycoprotein incorporation in the transgenic cell VLPs. Improved yield and immunogenicity of transgenic cell-produced VLPs may be achieved with the addition of further genetic elements into the *piggyBac *integron.

## Background

Human immunodeficiency virus type 1 (HIV-1) is responsible for the current infection of over 20 million people and the death of over 2 million living in sub-Saharan Africa [1]. Subtype C infections predominate in southern Africa and represent a large portion of the world wide infections [1], highlighting the need to develop a safe and effective vaccine based on Subtype C.

The HIV-1 precursor structural protein Pr55 Gag has been targeted as a potential candidate in vaccine studies as it is able to self-assemble and bud from a variety of cell systems to form non-replicating and non-infectious virus-like particles (VLPs) with good humoral and cell-mediated immune responses in animals. To date, Gag VLPs have been generated using various eukaryotic expression systems, but most often via the baculovirus-based transient protein expression system in insect cell cultures [2,3]. We have shown that baculovirus-derived HIV-1 Pr55 subtype C VLPs are able to elicit strong cellular immune responses in mice and baboons when administered as a boost to a HIV-1 *gag *DNA vaccine prime [4,5].

However, there are significant potential drawbacks to use of the baculovirus expression system: these include the necessity for constant maintenance of baculovirus stocks, the need for fresh batch infections to be made each time the product is required, and co-purification of recombinant baculovirus or baculovirus proteins with VLPs. The creation of a transgenic cell line for continuous culture and protein production may provide a way to bypass production issues arising with the use of baculovirus and to overcome potential safety issues with baculovirus particle contamination of VLP preparations.

The only reported attempts to transform *Spodoptera frugiperda *insect cells in culture include the random integration of an entire expression plasmid into the insect genome through recombination under antibiotic selection [6-11]. Transposon mutagenesis is an ideal alternative as it is based on a naturally occurring system in insect cells and has been extensively used to transpose many insect species [12,13]. The *piggyBac *transposable element has been widely studied and is favoured as a useful tool in insect transgenesis due to its simplicity of movement and often high frequency of transformation [14]. This class II element is derived from the cabbage looper moth *Trichoplusia ni *and is a member of the TTAA-target site-specific class of transposable elements [15]. It exclusively targets TTAA sites and duplicates this site upon insertion. The element is 2476 bp in length and encodes a single open reading frame (1.8 kbp) and terminates with 13 bp inverted terminal repeats (ITR). The ORF encodes a putative transposase (molecular weight of 64 kDa) which is responsible for the movement of the element [13]. Transgene integration into an insect genome is made possible by replacing the transposase ORF in the *piggyBac *vector with the transgene, while supplying the transposase *in trans *[13,15].

A number of whole insects from species spanning three orders [13,16] as well as non-insect species ranging from planarian to mammalian cells [17-20] have been transformed using the *piggyBac *vector system. This wide range of utility for this element makes it an attractive genetic tool. *piggyBac *transposons are favored over other elements as they are able to transpose large DNA fragments (9.6-14 kb) [20] making them suitable for applications in dual expression vectors designed to include selection markers, transcriptional activators or immune enhancer elements. Many pest species do not have transposons closely related to this element and the chance of re-transposition has been shown to be very low in several insect species studied [18,21-23].

No studies involving *piggyBac *transposon-mediated mutagenesis of cultured *Spodoptera *insect cell lines have been reported to date. Here we report the creation of transgenic *Spodoptera frugiperda *cell lines using the *piggyBac *system to express HIV-1 Gag protein, with the aim of developing a system for continuous production of HIV-1 Gag VLPs for vaccine studies. The immunogenicity of these transgenically expressed VLPs is compared to that of baculovirus produced VLPs in BALB/c mice.

## Methods

### Cell lines

The *Spodoptera frugiperda*-derived Sf21 cell line (Invitrogen) was maintained as a monolayer at 27°C in TC-100 insect medium (Sigma) supplemented with 10% (v/v) foetal bovine serum (FBS), 50 μg/ml neomycin, 69.2 μg/ml penicillin G and 100 μg/ml streptomycin. The *Spodoptera frugiperda*-derived Sf9 cell line (Invitrogen) was maintained as a shaking culture at 27°C, 140 rpm in SF900 II SFM media (Gibco) supplemented with 10 μg/ml gentamicin. The High 5™ cell line is derived from *Trichoplusia ni *(Invitrogen) and was maintained as a shaking culture in Express Five^® ^SFM media (Gibco) supplemented with 10 ug/ml gentamicin and 18 mM glutamine.

### Baculovirus produced Gag VLPs

The human codon-optimised HIV-1 subtype C *gag *DNA sequence used in this study was derived from the DNA vaccine plasmid pTHgagC [24,25]. The *gag *sequence used encodes a myristoylation signal responsible for directing the myristoylated Pr55 Gag protein to the host cell membrane where it embeds, aggregates and buds off as VLPs which are "coated" in host cell outer membrane [26,27]. Baculovirus produced Gag VLPs (BV) were generated in Sf9 cells using the Bac-to-Bac^® ^Baculovirus Expression Vector System [4,27,28]. Briefly, 1 × 10^6 ^Sf9 cells/ml were infected with baculovirus encoding human codon optimised HIV-1 subtype C *gag *under control of the *polh *promoter at a multiplicity of infection (MOI) of 2-10, and VLPs were harvested from the culture supernatant 72 h post infection.

### *piggyBac *plasmid constructs

The *piggyBac *minimal construct, pXLBacII has been previously described [14,15]. pXLBacII contains a multiple cloning site for insertion of a transgene between the 235 bp 3' ITR (Inverse Terminal Repeat) and 310 bp 5' ITR, but lacks the open reading frame encoding the *piggyBac *transposase which is necessary for transposition to occur. Instead, the *piggyBac *transposase open reading frame is encoded on a separate helper plasmid. We used as helper plasmids; pCasper-hs-orf (pCasper), in which the transposase gene is controlled by the *hsp*70 promoter, and pBSII-IE1-orf (pBSII-IE1), in which the transposase gene is controlled by the baculovirus immediate early (*ie*1) promoter. These plasmids have been described previously [15]. A transactivator plasmid (hr3IE1) to be included as a third plasmid in transfection, was supplied by K.J. Maragathavally [29,30]. Constructs harboring different regulatory elements were obtained from H. Zieler (see table 1) [31,32]. Constructs containing a 133 bp intron placed upstream or downstream to the *gag *gene (409-FOR, 410-FOR, 411-FOR, 412-FOR), were included for comparison of Gag expression levels with and without the inclusion of an intron [32].

**Table 1:** List of different regulatory elements used to design a set of *gag piggyBac *vector constructs.

Construct	Enhancer	Promoter	Intron upstream	Gene	Intron downstream	PolyA	Final pXLBacII construct
pHSP70		*Drosophila hsp*70		*gag*		SV40sti/polyA	pXLHSP70Gag

pIE1	*hr*5	Baculovirus Ac*M*NPV *ie*1		*gag*		(*Heliothis*) hel2polyA	pXLIE1Gag

pIE1	*hr*5	Baculovirus Ac*M*NPV *ie*1		Neo		hel2polyA	pXLNeo

pIE1-SV	*hr*5	Baculovirus Ac*M*NPV *ie*1		*gag*		SV40sti/polyA	pXLIE-SVGag

pActin		*Drosophila *actin 5C		*gag*		hel2-polyA	pXLActinGag

pActin-SV		*Drosophila *actin 5C		*gag*		SV40sti/polyA	pXLActin-SVGag

409-FOR	*hr*5	Baculovirus Ac*M*NPV *ie1*	**+**	*gag*		hel2polyA	pXL409Gag

410-FOR	*hr*5	Baculovirus Ac*M*NPV *ie*1		*gag*	**+**	hel2polyA	pXL410Gag

411-FOR		*Drosophila *actin 5C	**+**	*gag*		hel2-polyA	pXL411Gag

412-FOR		*Drosophila *actin 5C		*gag*	**+**	hel2-polyA	pXL412Gag

Hr3ieLuc	*hr*3	Baculovirus Ac*M*NPV *ie*1		*gag*			pXlHr3ieGag

pBSII-IE1-orf		Baculovirus Ac*M*NPV *ie*1		*gag*		pBSII-IE1-orf polyA	pXLBSII-IE1Gag

The promoters tested included *Drosophila hsp*70, Ac*M*NPV *ie*1 and *Drosophila *actin 5C [30,31] (column 3). The promoter enhancers used were *hr5 *[31] and *hr3 *[30] (column 2). Poly-adenylation (polyA) sequences included SV40sti/polyA, hel2polyA [31] and a polyA tail isolated from the pBSII-IE1-orf helper plasmid [14,15] (column 7). 409-FOR and 410-FOR are pIE1 vectors containing a 133 bp intron [32] (column 4 and 6) upstream or downstream of the *gag *gene, respectively. 411-FOR and 412-FOR are pActin vectors containing the intron upstream or downstream of the *gag *gene, respectively. Briefly, different promoters, enhancers, polyA sequences or the intron were removed from the construct described in column 1 and coupled to *gag *in the transposable pXLBacII vector [14,15] (column 8).

### *piggyBac *construct design

The human codon-optimised, myristoylated HIV-1 subtype C *gag *gene was coupled to different regulatory elements and cloned into the *piggyBac *minimal construct pXLBacII, to be used in transfections (see table 1).

A digenic construct was designed to harbor the neomycin gene coupled to the *gag *gene (*NeoGag*) to enable antibiotic selection of transgenic cells. This construct was created by cloning the *ie*1-*gag*-polyA fragment from the pXLBSII-IE1Gag construct, downstream of the *neo *gene in pXLNeo (see table 1).

### Transfection of *Spodoptera frugiperda *cells

Each of the pXLBacII constructs containing the *gag *or *NeoGag *gene, together with one of the helper plasmids, was co-transfected into Sf21 cells with Cellfectin (Invitrogen) for 5 hours. DNA amounts of 1 to 5 μg were used to transfect 1.5 × 10^6 ^Sf21 cells per 2 ml, and the ratios of the transgene to helper plasmids were varied from 1:1, 7:1 and 1:2 [33,34]. Two different helper plasmids, pCasper and pBSII-IE1, were tested for differences in transgene integration as assessed by subsequent Gag expression levels. The IE1 transactivator (hr3IE1) was included during transfection at a tenth of the total DNA amount to determine its potential to improve promoter activity or transposition frequency [29,30]. A comparison of transformability and transgenic Gag expression levels was conducted between three different insect cell lines, Sf21, Sf9 and High 5™.

### PCR detection of the transgene and determination of the integration event

**Figure 1:**
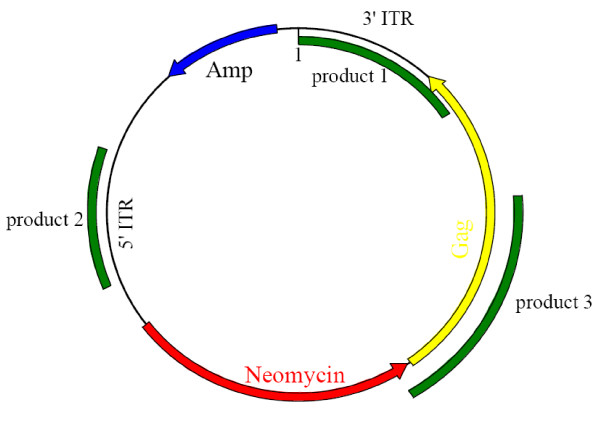
**pXLBacII*NeoGag *vector**. PCR products and amplification sites for the primers depicted in table 2 are indicated on the vector.

DNA was extracted from ± 5 × 10^6 ^transgenic insect cells using the Dneasy Tissue Extraction kit (Qiagen) and subjected to PCR screening. Primers were designed to amplify the transgenes (*gag *or neomycin) to assess their presence and stability over several passages (see table 2). To determine whether the entire *piggyBac *vector or only the transgene integrated into the insect cell genome, primers were designed to amplify different sites on the pXLBacII *NeoGag *vector. See table 2 for the primer sequences and Figure 1 for their respective positions on the vector. PCR screening was carried out on a number of transgenic insect cell lines expressing Gag under the control of various regulatory elements (table 3).

**Table 2:** Primer sequences used in the screening of transgenic insect cell lines expressing Gag protein.

Primer pair	Product
sense CTAAATTGTAAGCGTTAATATTTTG antisense GGTAGCGACCCCCTCAGCCAATAAGAATTC	Product 1 (3'ITR and 3' end of *gag*)

sense: GGGTTAATCTAGCTGCATCAGGATC antisense: GAGCGCACGAGGGAGCTTC	Product 2 (5' ITR)

sense: CTGGGATGGGTGGGTTGCTGG antisense:CTTCTGAGAGCTCGATCGATAC	Product 3 (Junction between neomycin and *gag*)

sense ATGGGTGCTCGCGCATCTATCC antisense ATTCTTGGCTGAGGGGGTCGC	Gag

sense GCGGTACCATGATTGAACAAG antisense CGGAGCTCTCAGAAGAACTCG	Neomycin

sense CAGTTACCAATGCTTAATCAG antisense GTATGAGTATTCAACATTTCCG	Ampicillin

**Table 3:** PCR screening results of transgenic insect cell lines.

Construct	Product 1	Product 2	Product 3	*gag*	Neo	Amp
pXL*NeoGag *passage 15 in Sf21 cells	+	+	+	+	+	+

pXL*NeoGag *passage 26 in Sf21 cells	+	+	+	+	+	+

pXL*NeoGag *passage 23 in Sf9 cells	X	+	+	+	+	+
**passage 7**				**+**		
**passage 13**				**+**		
**passage 93**				**+**		

pXLIE-SVGag passage 16 in Sf21 cells	X	+	X	+	X	+

pXlHr3ieGag passage 9 in Sf21 cells	X	+	X	+	X	+

pXLHSP70Gag passage 6 in Sf21 cells	X	+	X	+	X	+

Screening was carried out using primers complementary to different sections of the *pXLBacIINeoGag *vector. The presence of a PCR product is indicated with a plus (+) and absence with a cross (X). Confirmation of stable integration up to 93 passages was indicated by performing PCR amplification on several passages of pXL*NeoGag *in Sf9 cells (see bold font).

### Determination of whether Gag mRNA is transcribed from an integrated transgene or from a persisting non-integrated *piggyBac *vector plasmid

To confirm that the pXLBacII vector is absent from the transgenic insect cell culture, and hence not contributing to Gag expression, rolling circle amplification (RCA, GE Healthcare, Amersham) was carried out on DNA isolated from harvested cells. RCA is based on a bacteriophage ϕ29 DNA polymerase that exponentially amplifies single- or double-stranded circular DNA templates by rolling circle amplification. To confirm the absence of inhibitory elements, the isolated DNA sample was spiked with 0.7 ng (± 7 × 10^7 ^copies) to 0.007 ng (± 7 × 10^5 ^copies) of the pXLBacII*NeoGag *vector and amplified according to the manufacturer's protocol (GE Healthcare, Amersham). Amplified products were subsequently digested with Mlu1 (which cuts twice within the *NeoGag *region) and analysed on a 0.8% agarose gel with ethiduim bromide staining.

### Screening for optimal protein expression using ELISA

To determine which method of transfection and which regulatory variable generated a cell line capable of secreting optimal Gag yields, cell supernatants were screened for the p24 component of Gag using a p24 antigen ELISA kit (Vironostika, Biomeriuex) according to the manufacturer's instructions.

### Selection assay

The *NeoGag *construct, together with the pCasper helper, was transfected into Sf21 cells. Selection using 1 mg/ml geneticin (G418, Sigma) [7,6] began two days post transfection. Transgenic cells were exposed to geneticin after 40 passages (20 weeks) and examined for viability using an inverted light microscope (Carl Zeiss "Axioskop"). Non-transgenic cells were exposed to the antibiotic and used as a negative control.

### VLP isolation

*piggyBac *(*PB*)-produced VLPs were harvested from 1.5 × 10^9 ^transgenic *NeoGag *insect cells and baculovirus produced (BV) VLPs were harvested from 24 × 10^7 ^baculovirus infected cells. Cell culture supernatants were clarified by low speed centrifugation at 1000 g for 10 minutes. VLPs in these supernatants were pelleted at 4°C by ultracentrifugation in a Beckman SW32ti rotor at 26 000 rpm for 90 minutes. The pellets were then resuspended in phosphate buffered saline (1 × PBS, pH 7.4) and fractionated by centrifugation using a 10-50% Optiprep^® ^(Sigma) step gradient at 26 000 rpm in a Beckman SW32ti rotor for 4 hrs at 4°C. Three light-scattering bands observed at the 10/20%, 20/30% and 30/40% Optiprep^® ^interphases (identical for both *PB *and BV VLP preparations) were collected and pooled. VLPs were pelleted, after dilution of the Optiprep^® ^with PBS, at 26 000 rpm for 90 minutes in a SW32ti rotor then resuspended in l × PBS. Both *PB *and BV VLP preparations were confirmed negative for the presence of endotoxins (QCL-1000^® ^Chromogenic LAL Kit, Cambrex).

### Electron microscopy

Gradient purified samples were adsorbed onto carbon coated copper grids, stained with 2% uranyl acetate and visualized with a LEO 912 transmission electron microscope.

### Immunoblotting and quantification of *PB *and BV VLPs

The banding patterns of purified *PB *and BV VLP samples were analysed on Coomassie stained gels and anti-p24 western blots. Aliquots of purified *PB *and BV VLPs were fractionated by electrophoresis through a 10% denaturing SDS polyacrylamide gel and either stained with 0.1% Coomassie Blue stain to assess the relative purity of the preparation, or blotted to a nitrocellulose membrane (Nitrobond, Osmonics Inc.) using a semi-dry electroblotter (Hoefer) for 1.5 h at 15 V. Pre-stained molecular weight standards (PageRuler, Fermentas) and a serial dilution of a HIV-1 Pr41 Gag positive control (41 kDa; Quality Biological Inc., USA) was included on the gel. Membranes were probed with 1:10 000 dilution of anti-p24 rabbit antiserum (ARP432, NISBC Centralised Facility for AIDS reagents, MRC, UK) followed by a 1:5000 dilution of anti-rabbit alkaline phosphatase--conjugated secondary antibody (Sigma). Membranes were developed with Nitro blue tetrazolium chloride/5-bromo-4-chloro-3-indolyl phosphate (NBT/BCIP, Roche).

Gag concentrations were determined by comparing calculated densities of the Pr55 bands in experimental and control samples using gel imaging software (Syngene). VLP concentrations were determined by calculating densities of the Pr55 band on western blots rather than using p24 ELISA quantification, as the Pr55 content is a more reliable indicator of the actual VLP concentration in the sample than the p24 content. VLP preparations were formulated with 15% trehalose to 100 ng Gag/100 μl PBS then stored at -70°C.

Baculovirus gp64 envelope glycoprotein content of *PB *and BV VLPs was assessed by western blot analysis as described above using a 1:10 000 dilution of anti-baculovirus envelope gp64 antibody (Clone: AcV5, eBioscience), followed by a 1:5000 dilution of anti-mouse alkaline phosphatase--conjugated secondary antibody (Sigma). Membranes were developed with NBT, as above.

### Immunization of mice and detection of cellular immune responses

We compared the immunogenicity in BALB/c mice of *PB *VLPs and BV VLPs when given alone, and their ability to boost a response to a prime with the matched HIV-1 subtype C DNA vaccine, pTHgagC [24,25,35]. VLPs (100 ng Gag protein in 100 μl PBS) and DNA (100 μg DNA in 100 μl PBS) were given to groups of female BALB/c mice (5 mice per group) via the intramuscular route with 50 μl injected into each quadriceps muscle. To test responses to VLPs alone, mice were inoculated with a single dose of either *PB *Gag VLPs or BV Gag VLPs on day 0, and spleens from VLP-inoculated mice were harvested on day 12. To assess VLP boosting of a DNA prime, mice were given a single dose of pTHGagC on day 0 and boosted with a single dose of either *PB *Gag VLPs or BV Gag VLPs given on day 28 and spleens were harvested on day 40. Mice vaccinated with pTHGagC on day 0 without any boosting on day 28 were used as a DNA primed, unboosted control group and their spleens were likewise harvested on day 40. All procedures were carried out according to guidelines and with approval of the UCT Animal Research Ethics Committee. Cell mediated immune responses were determined using splenocytes in Interferon gamma (IFN-γ) and interleukin 2 (IL-2) ELISPOT assays (BD Pharmingen) with Gag CD8 peptide (AMQMLKDTI), and Gag CD4 (13) and Gag CD4 (17) peptides (NPPIPVGRIYKRWIILGLNK and FRDYVDRFFKTLRAEQATQE, respectively) as previously described [27].

## Results

### Transgene expression

p24 ELISA was used to screen the culture supernatants from different transgenic cell lines maintained in culture. All preliminary experiments were carried out in Sf21, Sf9 and High 5™ cell lines in order to confirm that these lines were amenable to *piggyBac *transformation and subsequent Gag VLP expression. For reasons of practical handling, all further experiments were carried out using only Sf21 cell lines, for small scale amplification, and Sf9 cell lines, for large scale amplification (Sf9 cells being more suitable to this because they were maintained as a shaking culture versus Sf21 cells that were maintained as a monolayer culture). Sf21 cells and Sf9 cells expressed the Gag protein at similar levels for each construct tested. The highest p24 expression level obtained was from cell lines transgenic for the *gag *gene coupled to the *ie*1 promoter and the pBSII-IE1 polyA tail (derived from pXLBacSII-IE1Gag). Expression from these cell lines ranged between 100-1000 pg per 1.5 × 10^6 ^cells. Previous work suggested that inclusion of an intron adjacent to a gene can stimulate its transcription and possibly enhance protein expression levels [32]. No significant expression variations were observed when the same intron was used in this study. No p24 expression was evident when the *gag *gene was placed under the *hsp*70 or actin 5C promoters. Further investigation into transgenic *hsp*70-*gag *cell lines confirmed the integration of the construct (see table 3) ruling out the possibility that no transgene integration had occurred.

Inclusion of a third plasmid expressing a transactivator is known to improve protein expression levels by enhancing promoter activity [30] or by increasing transpositional frequency of the element [29]. No significant variation in Gag expression was observed when the IE1 transactivator was included at transfection. Use of different helper plasmids and transfection ratios did not affect the expression either.

### Persistence of expression

Transgenic Sf21 and Sf9 cell lines continued to express Gag protein at constant levels (as determined using p24 ELISA) for at least 100 cell passages. Table 3 and 4 indicate the PCR amplification results determined for several transgenic insect cell lines. PCR amplification of the *gag *transgene from insect cell genomic DNA remained positive after 93 cell passages. PCR amplification of the neomycin transgene from insect cell genomic DNA remained positive for 30 cell passages and was not tested further. Exposure of the Sf21 *NeoGag *cells to geneticin at passage 40 had no effect on their survival, which was in contrast to the non-transgenic control cells that lost viability within three to four days. The geneticin was not administered prior to passage 40 indicating that it is not a requirement for stable integration. These results strongly indicate stable integration and expression of the transgene over many cell generations.

**Table 4:** Continuous expression of p24 protein from several transgenic insect cell lines as determined by ELISA.

Cell line and passage number	*gag *transgene presence (PCR)	Gag expression: pg p24/ml culture supernatant (1.5 × 10^6 ^cells)
pXL*NeoGag *passage 7 in Sf9 cells	+	129.87 pg/ml

pXL*NeoGag *passage 13 in Sf9 cells	+	129 pg/ml

pXL*NeoGag *passage 23 in Sf9 cells	+	75.47 pg/ml

pXL*NeoGag *passage 93 in Sf9 cells	+	606.6 pg/ml

The presence and active transcription of the *gag *transgene in the Sf9 cell line pXL*NeoGag*, was confirmed over several passages using PCR and p24 ELISA screening, respectively.

### Integration of the *piggyBac *vector

To examine the nature of the integration event that had occurred, PCR screening of the *piggyBac *vector and the transgenes was carried out on several transgenic insect cell lines. Table 3 indicates the absence or presence of the PCR products in selected cell lines (refer to table 2). Product 1 is an amplification of the 3' end of the *gag *gene and the 3' *piggyBac *border (3' ITR), product 2 is an amplification of the 5' *piggyBac *border (5' ITR). Product 1 and 2 would be absent if canonical *piggyBac *transposon mutagenesis had occurred. Product 3 is the 3' end of the neomycin gene and the 5' end of the *gag *gene, providing evidence of transgene integrity. The majority of results indicate that the plasmid was linearised at the 3' ITR prior to integration of the entire intact *piggyBac *plasmid, possibly through a recombination mechanism unrelated to tranposition. A study in *Aedes aegypti *using *piggyBac *transformation revealed similar non-canonical integration of sequences from both donor and helper plasmids [22,36]. No evidence of helper plasmid integration was evident in our study as confirmed by PCR screening for the *hsp*70 promoter in transgenic cell lines transfected with the pCasper helper plasmid (data not shown). However, these results are inconclusive in predicting whether recombination or transposon insertion occurred, as single insect cell clones were not isolated post transfection and mixed populations may exist.

### Gag expression is not due to persisting non-integrated plasmid

**Figure 2:**
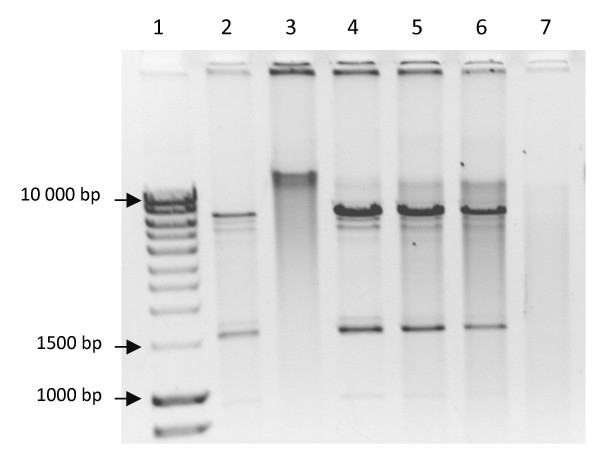
**RCA amplification carried out on DNA extracted from *NeoGag *expressing transgenic cells**. The RCA product was digested with Mlu1 and visualised on an 0.8% agarose gel. Lane 1- Fermentas Bioline hyperladder 1 with sizes indicated in basepairs. Lane 2- positive control (pXLBacII*NeoGag*). Lane 3- Sf21 *NeoGag *genomic DNA. Lane 4- Sf21 *NeoGag *genomic DNA spiked with 7 ng of the positive control. Lane 5- Sf21 *NeoGag *genomic DNA spiked with 700 pg of positive control. Lane 6- Sf21 *NeoGag *genomic DNA spiked with 70 pg of positive control. Lane 7- Sf21 *NeoGag *genomic DNA spiked with 7 pg of positive control.

Rolling circle amplification (RCA) was carried out on DNA extracted from transgenic Sf21 *NeoGag *cells to eliminate the possibility of transient Gag expression from a persistent non-integrated circular *piggyBac *vector. As seen in Figure 2, lane 3, no circular DNA that resembled the pXLBacII*NeoGag *plasmid (lane 2) was detected in the transgenic DNA sample. The minimum amount of pUC19 DNA that can be amplified using RCA is 1 pg (± 3 × 105 copies) (RCA instruction manual, GE Healthcare, Amersham). To discount the possibility that non-amplification could be attributed to a low copy of the circularised DNA or the presence of inhibitors in the isolated DNA sample, transgenic insect DNA extract was spiked with 10-fold dilutions of the pXLBacIINeoGag plasmid prior to amplification. A minimum of 70 pg (± 7 × 10^6 ^copies) of spiked construct was detectable. No amplification of 7 pg of spiked plasmid (± 7 × 10^5 ^copies) occurred. This copy number approaches the limits of detectability for the RCA assay. Together these results suggest that absence of detectable RCA amplification in transgenic Sf21 *NeoGag *cells is not due to inhibitor presence but is rather due to absence of non-integrated plasmid in the samples.

### Size and morphology of *PB *and BV VLPs

**Figure 3:**
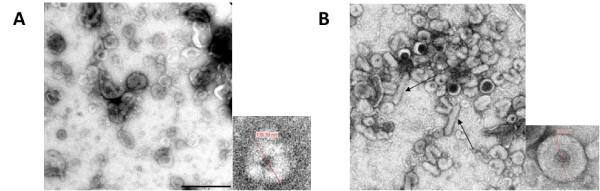
**Electron micrographs of *PB *and BV VLPs**. A: *PB *VLPs, scale bar 500 nm. B: BV VLPs, same scale. Arrows show contamination by co-purified recombinant baculovirus in the BV sample. Insets: individual particles with diameter shown. *PB *VLP: 139 nm; BV VLP: 129 nm.

EM analysis of the three protein bands isolated from the *PB *and BV gradients revealed particles of size 120-150 nm in the 10/20% and 20/30% Optiprep^® ^interphases. VLP size range and morphology resembled that of VLPs previously reported [3,4,27]. *PB *VLPs and BV VLPs were similar in size and shape although BV VLPs appeared more defined and compact than the *PB *VLPs (see Figure 3).

### Western blot immunodetection and quantification of *PB *and BV VLPs

**Figure 4:**
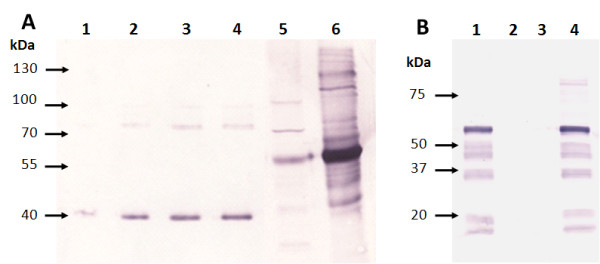
**Western blot immunodetection of *PB *and BV VLPs**. A: Anti-p24 western blot. Lane 1-1.2 ng Pr41 std. Lane 2-2.4 ng Pr41 std. Lane 3-4.8 ng Pr41 std. Lane 4-7.2 ng Pr41 std. Lane 5- purified *PB *VLPs from the 20/30% Optiprep^® ^interphase (22 ug of total soluble protein loaded). Lane 6- purified BV VLPs from the 20/30% Optiprep^® ^interphase (0.68 ug of total soluble protein loaded). B: Anti-gp64 western blot. Lane 1- Supernatant from cells infected with negative baculovirus (no *gag *insert). Lane 2- purified *PB *VLPs from the 10/20% Optiprep^® ^interphase. Lane 3- purified *PB *VLPs from the 20/30% Optiprep^® ^interphase. Lane 4- purified BV VLPs from the 20/30% Optiprep^® ^interphase. The respective sizes from markers run on the same gels are indicated in kDA on the left of both blots.

Purified Gag VLPs extracted from the transgenic cell culture and from recombinant baculovirus infected cells were analysed and quantified on an anti-p24 western blot using densitometry. An identical Pr55 Gag band was detected in both *PB *and BV VLP preparations (Figure 4A). Quantification of purified *PB *VLPs on western blots against a serial dilution of a HIV-1 Pr41 positive control indicated yields of 1-2 ng per 1 × 10^6 ^Sf9 cells, at least 1000 times lower than yields obtained for BV expressed VLPs. Anti-gp64 western blot results show the absence of gp64 envelope protein in *PB *VLP samples and its presence in BV VLP samples (Figure 4B).

**Figure 5:**
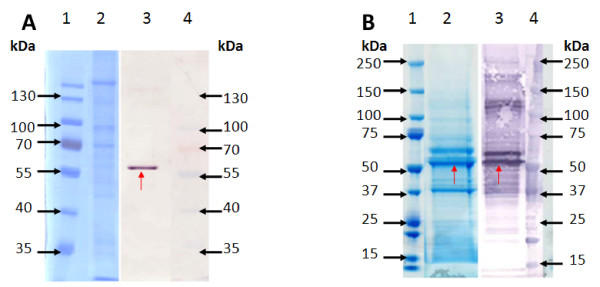
**Comparison of *PB *and BV VLP banding pattern differences as visualised on a Coomassie stained gel and western blot**. Coomassie Blue stained 10% denaturing polyacrylamide gels are shown on the left of 5A and 5B and anti-p24 stained western blots are shown on the right of 5A and 5B. Protein size markers on gels and blots are indicated in kDA. Red arrows indicate the Gag Pr55 band. A- Purified *PB *VLPs. Lanes 1 and 4 - MW marker. Lane 2- Coomassie Blue stained *PB *VLP preparation (30 ul of purified sample, corresponding to 6 pg p24). Lane 3- *PB *VLP preparation detected with anti-p24 antibody (30 ul of purified sample, corresponding to 6 pg p24). B- Purified BV VLPs. Lanes 1 and 4 - MW marker. Lane 2- Coomassie Blue stained BV VLP preparation (28 ul of purified sample, corresponding to 170 ng p24). Lane 3- BV VLP detected with anti-p24 antibody (28 ul of purified sample, corresponding to 17 ng p24).

As seen in Figure 5, the Pr55 Gag protein of the BV VLP sample was readily detected on a Coomassie stained gel as well as on an anti-p24 western blot. The Pr55 Gag protein of the *PB *VLP sample was detected at the same protein size location on the anti-p24 western blot, but was not detectable against the background on the Coomassie stained gel, as was expected given the low p24 concentration of the loaded sample).

### Immunogenicity of VLPs

**Figure 6:**
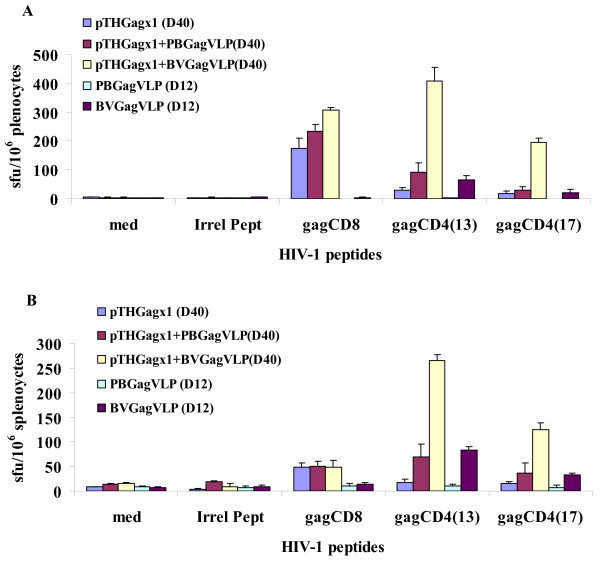
**Elispot results from *PB *and BV immunised mice**. IFNγ (A) and IL-2 (B) ELISPOT analysis of Gag CD8+ and CD4+ T cell responses on day 12 (D12) after vaccination of mice with *PB *or BV VLPs or responses on day 40 (D40) for mice primed with pTHgagC on day 0 then boosted on day 28 with *PB *or BV VLPs. Splenocytes prepared from spleens combined from five mice per group were used in IFN-γ or IL-2 ELISPOT assays with the indicated *gag *peptides or irrelevant peptide (irrel pept) or in the absence of peptide (med) as indicated. Bars are the mean number of spots of triplicate reactions for 10^6 ^splenocytes with indicated standard deviation of the mean.

It has previously been shown that BV Gag VLPs enhance a cellular immune response in mice when used as a boost to a pTHgagC DNA prime [4,27]. To assess whether the *PB *VLPs elicited a similar response in the absence of baculovirus elements, mice were inoculated with equal quantities of either BV or *PB *VLPs. Because the study reported here was designed in proof of concept, we conducted only a standard heterologous prime-boost immunogenicity experiment, in which we compared the boost potential of *PB *VLPs versus BV VLPs, using the same dosage which our group had previously found optimal when using BV VLPs to elicit a boosted immunological response in DNA inoculated mice: that is, 100 ng of HIV-1C VLPs administered as 2 × 50 ng inoculated into each quadriceps muscle alone or as a boost to DNA primed mouse at day 28 (ms. submitted; S Pillay, A Meyers, EG Shephard, A-L Williamson, EP Rybicki). IFN-γ and IL-2 ELISPOT analysis (Figure 6) showed that *PB *VLPs did not induce an immune response nor did they boost an immune response to a DNA vaccine prime. In contrast the ELISPOT assays detected a low magnitude of GagCD4(13)-specific IFNγ and IL-2 producing cells but no GagCD8-specific cells in response to BV VLP vaccination. These BV VLPs boosted pTHGagC responses. A 1.8 fold increase in the response to the GagCD8 peptide was detected in the IFN-γ ELISPOT assay. The low cumulative response to the GagCD4(13) and GagCD4(17) peptide of 50 IFN-γ sfu/10^6 ^splenocytes induced by pTHGagC increased 12 fold to 602 sfu/10^6 ^splenocytes when pTHgagC primed mice were boosted with these BV VLPs. No boost of the DNA vaccine induced GagCD8 response was detected in IL-2 assays. Although pTHgagC did not induce Gag-specific CD4+ T cells that produced IL-2, a high cumulative magnitude of 390 sfu/10^6 ^splenocytes Gag-specific CD4(13) and GagCD4(17) T cells producing IL-2 was detected in response to a DNA prime BV VLP boost.

## Discussion

Gag epitopes elicit robust CTL responses that effectively control HIV-1 viral load in the early phases of HIV-1 infection, and Gag is therefore thought to be highly suitable as a vaccine candidate to elicit CTL [3]. HIV-1 Gag VLPs, in particular, are widely accepted as being strongly immunogenic particulate antigens that stimulate good CTL responses in prime/boost vaccination strategies [3-5,27,37]. Baculovirus production of HIV-1 Gag VLPs is a well documented method for generating immunogenic HIV-1 particles, but contamination of VLPs with co-purified baculovirus particles (Figure 3) is not favourable for their subsequent use in vaccine studies. To address this problem and the problem of routine maintenance of infectious stocks, we utilised *piggyBac *transposon mutagenesis as a novel method of generating *gag*-transgenic insect cell lines for continuous production of HIV-1 Gag VLPs. Expression of Gag VLPs from these transgenic cell lines proved stable for at least 100 cell passages: this is a novel result, which may have valuable implications in future HIV and other vaccine work.

Using immunodetection and EM we verified that Gag VLPs were secreted from transgenic insect cell lines. However, the yields were low (at least 1000 times lower than from the baculovirus production system) and therefore we tested the ability of various regulatory elements to improve protein expression of the *gag *transgene. No improvement in Gag protein expression was noted when we cloned hr5 and hr3 baculovirus-derived enhancer elements [38] or introns [32] into the *piggyBac *construct, nor when we included the transactivator during transfection [30,39]. Different molar transposase-to-transposon ratios did not affect Gag expression levels, which also confirms previous observations that the *piggyBac *system does not demonstrate overproduction inhibition [20]. In preliminary experiments, we noted that the *hsp*70 or actin 5C promoters were not active in the *Spodoptera *transgenic cell lines, and so no further work was done with these constructs. The *Bombyx mori *actin 3C promoter and *Drosophila *ubiquitin promoter have proven active in a variety of insect species [13] and should be evaluated in *Spodoptera *insect cell culture as an alternative means to possibly improve transgene expression.

Although Gag VLP yields obtained in this preliminary study were low, we are confident that there are several approaches that can be employed to improve protein expression yields. Inclusion of a Gal4 DNA binding domain as an N-terminal fusion to the transposon has been shown to increase the number of transposition events [40] which in turn can result in improved protein expression levels. It has been shown recently that the translation enhancer activity of 5'-UTR *pol *(un-translated region of the nucleopolyhedrovirus polyhedron gene) is able to improve transgene expression when placed upstream to the promoter [41]. Poor transgene expression could be attributed potentially to the integration of the gene into an unfavourable genomic site such as a silent heterochromatin region or near to unfavourable transcription enhancers. This can be overcome by directing the transposon construct to a targeted site using the *Gal4/UAS *or *FLP/FRT *system [40,42,43], or by surrounding the transgene with an insulator [44]. Transposon constructs can be designed to harbour bidirectional promoters that drive simultaneous expression of the transgene and a strong artificial transcriptional activator [45], leading to improved transgene expression levels.

However, it is possible that constitutive production of Gag protein is toxic to the cell, so only low expressers survive. In this case, inducible expression systems could be explored.

We conducted a comparative study in mice to assess the relative immunogenicity of baculovirus-produced VLPs versus *piggyBac *transgenically expressed VLPs. While BV VLPs were able to induce a good CD4 immune response in mice when administered as a boost to a DNA prime, *PB *VLPs, on the other hand, elicited no immune response, showing that Gag VLPs are not intrinsically as highly immunogenic as previously thought, and that baculovirus-derived elements probably enhance Gag VLP immunogenicity. Baculovirus-expressed Gag VLPs include trace amounts of insect cell and baculoviral contaminants (lipids, nucleic acids and proteins) that are not efficiently removed during purification, as well as incorporated baculovirus envelope proteins. Deml *et al *proposed that these contaminating components act as "danger signals" that can activate an innate immune response [3,26]. It has also been shown that VLPs isolated from yeast [46] as well as from the baculovirus expression system [3] contain host cellular contaminants capable of stimulating human antigen presenting cells (APC) by up-regulating the maturation of cytotoxic T lymphocyte (CTL) markers and inducing cytokine secretion. Although it was beyond the ambit of the current study, it would be interesting to compare the relative ability of *PB *VLPs and BV VLPs to stimulate dendritic cells, as this could provide insight into the observed differences in immunogenicity between *PB *and BV VLPs in the study reported here.

Baculovirus has been shown to transduce mammalian cells, which could lead to adjuvanting of immune responses [47,48]. A recent study showed that intranasal inoculation of mice with a wild-type baculovirus induces a strong innate immune response, which protects mice from a lethal challenge of influenza virus [49]. Cellular uptake of baculovirus and subsequent immune response enhancement may be due largely to the presence of the IFN-stimulatory baculovirus surface envelope glycoprotein gp64, which is responsible for host cell receptor binding and membrane fusion during viral entry by endocytosis [50]. In particular, gp64 protein is known to incorporate into the outer surface of baculovirus expressed Gag VLPs [3,26,47,51]. This has been additionally demonstrated in the current study, where both BV VLPs and *PB *VLPs are "coated" in host cell outer membrane, but gp64 would be available for incorporation only into the BV VLPs (Figure 4). We observed that while *PB *VLPs were similar in size and morphology to BV VLPs, as seen by EM, they did not appear to be as compact or sharply defined in shape as BV VLPs (Figure 3). Since *PB *VLPs lacked baculovirus gp64 incorporation into the VLPs outer membrane coating, the more defined shape of the BV VLPs compared to that of the *PB *VLPs may be accounted for by the incorporation of gp64 into the BV VLP outer membrane. It is likely that incorporation of gp64 onto the surface of baculovirus expressed VLPs facilitates BV VLP uptake into APC by promoting membrane fusion between BV VLPs and host cells, thereby enhancing the resultant immune response to VLP Gag antigens conditioned by APCs. Transgenically produced VLPs lack gp64 and this may result in less efficient cellular uptake of VLPs, with resultant lower immunogenicity.

The use of molecular adjuvants or incorporation of gp64 onto VLP surfaces could be utilised to improve the immunogenicity of these particles expressed from transgenic insect cell lines. Shi *et al*. [33] demonstrated the use of *piggyBac *transposon vectors to transiently express two gene products. In this system, the two genes were placed under the control of bidirectional promoters which in turn were enhanced by a single enhancer element. Such dual expression systems could be used to co-express immune enhancer elements [52] or immunogenic baculovirus elements together with HIV-1 Gag VLPs to improve VLP immunogenicity.

Once a cell line has been established that transgenically expresses Gag VLPs at a high level and with enhanced immunogenicity features, permanent stabilisation of the transgene in *piggyBac-*transformed insect cell lines would be carried out by transgene integration site elimination. It has been demonstrated that elimination of the *piggyBac *transposon integration sites adjacent to the integrated transgene renders the element immobile to further transposase exposure [43,53].

## Conclusion

This study serves as a basis to indicate the potential of a transgenic insect cell expression system as an alternative to the baculovirus-insect cell production system. Stably transformed cells produced VLPs reliably over 100 passages; purification of VLPs was also easier than in the baculovirus system due to lack of heterologous virus particles. However, further work is needed to improve VLP expression levels and their immunogenicity.

## Authors' contributions

AL participated in the design of the study, carried out most of the experimental work and drafted the manuscript. FT conceived the study, participated in the design of the study, and helped in drafting and revising the manuscript. ES performed the immunological experiments described in the study and participated in experimental design. MF provided the *piggyBac *transposons and protocols and helped with the ms. A-LW was overall study director and obtained the funding, and helped in drafting the ms. EPR participated in study design and supervision and helped to revise the manuscript. All authors read and approved the final version.

## References

[B1] UNAIDS (2007). UNAIDS AIDS Epidemic Update: November 2007

[B2] Wagner R Deml L Schirmbeck R Niedrig M Reimann J Wolf H Construction, expression, and immunogenicity of chimeric HIV-1 virus-like particles Virology 1996 10 128 140 10.1006/viro.1996.0293 8659105

[B3] Deml L Speth C Dierich MP Wolf H Wagner R Recombinant HIV-1 Pr55 gag virus-like particles: potent stimulators of innate and acquired immune responses Mol Immunol 2005 10 259 277 10.1016/j.molimm.2004.06.028 15488613

[B4] Jaffray A Shephard E van HJ Williamson C Williamson AL Rybicki EP Human immunodeficiency virus type 1 subtype C Gag virus-like particle boost substantially improves the immune response to a subtype C gag DNA vaccine in mice J Gen Virol 2004 10 409 413 10.1099/vir.0.19396-0 14769898

[B5] Chege GK Shephard EG Meyers A van HJ Williamson C Lynch A Gray CM Rybicki EP Williamson AL HIV-1 subtype C Pr55 gag virus-like particle vaccine efficiently boosts baboons primed with a matched DNA vaccine J Gen Virol 2008 10 2214 2227 10.1099/vir.0.83501-0 18753231

[B6] McCarroll L King LA Stable insect cell cultures for recombinant protein production Curr Opin Biotechnol 1997 10 590 594 10.1016/S0958-1669(97)80034-1 9353223

[B7] Joyce KA Atkinson AE Bermudez I Beadle DJ King LA Synthesis of functional GABAA receptors in stable insect cell lines FEBS Lett 1993 10 61 64 10.1016/0014-5793(93)80439-2 8243667

[B8] Hollister J Grabenhorst E Nimtz M Conradt H Jarvis DL Engineering the protein N-glycosylation pathway in insect cells for production of biantennary, complex N-glycans Biochemistry 2002 10 15093 15104 10.1021/bi026455d 12475259 PMC3612895

[B9] Breitbach K Jarvis DL Improved glycosylation of a foreign protein by Tn-5B1-4 cells engineered to express mammalian glycosyltransferases Biotechnol Bioeng 2001 10 230 239 10.1002/bit.1112 11400096 PMC3644115

[B10] Aumiller JJ Hollister JR Jarvis DL A transgenic insect cell line engineered to produce CMP-sialic acid and sialylated glycoproteins Glycobiology 2003 10 497 507 10.1093/glycob/cwg051 12626399 PMC3612900

[B11] McLachlin JR Miller LK Stable transformation of insect cells to coexpress a rapidly selectable marker gene and an inhibitor of apoptosis In Vitro Cell Dev Biol Anim 1997 10 575 579 10.1007/s11626-997-0101-7 9282319

[B12] Ding S Wu X Li G Han M Zhuang Y Xu T Efficient transposition of the piggyBac (PB) transposon in mammalian cells and mice Cell 2005 10 473 483 10.1016/j.cell.2005.07.013 16096065

[B13] Handler AM Use of the piggyBac transposon for germ-line transformation of insects Insect Biochem Mol Biol 2002 10 1211 1220 10.1016/S0965-1748(02)00084-X 12225912

[B14] The *piggyBac* 2008 http://piggybac.bio.nd.edu/

[B15] Fraser MJ Cary L Boonvisudhi K Wang HG Assay for movement of Lepidopteran transposon IFP2 in insect cells using a baculovirus genome as a target DNA Virology 1995 10 397 407 10.1006/viro.1995.1422 7645244

[B16] Bossin H Furlong RB Gillett JL Bergoin M Shirk PD Somatic transformation efficiencies and expression patterns using the JcDNV and piggyBac transposon gene vectors in insects Insect Mol Biol 2007 10 37 47 10.1111/j.1365-2583.2006.00693.x 17257207

[B17] Li X Harrell RA Handler AM Beam T Hennessy K Fraser MJ Jr piggyBac internal sequences are necessary for efficient transformation of target genomes Insect Mol Biol 2005 10 17 30 10.1111/j.1365-2583.2004.00525.x 15663772

[B18] Condon KC Condon GC Dafa'alla TH Forrester OT Phillips CE Scaife S Alphey L Germ-line transformation of the Mexican fruit fly Insect Mol Biol 2007 10 573 580 17894556 10.1111/j.1365-2583.2007.00752.x

[B19] Mitra R Fain-Thornton J Craig NL piggyBac can bypass DNA synthesis during cut and paste transposition EMBO J 2008 10 1097 1109 10.1038/emboj.2008.41 18354502 PMC2323262

[B20] Wilson MH Coates CJ George AL Jr PiggyBac transposon-mediated gene transfer in human cells Mol Ther 2007 10 139 145 10.1038/sj.mt.6300028 17164785

[B21] Handler AM McCombs SD The piggyBac transposon mediates germ-line transformation in the Oriental fruit fly and closely related elements exist in its genome Insect Mol Biol 2000 10 605 612 10.1046/j.1365-2583.2000.00227.x 11122469

[B22] O'Brochta DA Sethuraman N Wilson R Hice RH Pinkerton AC Levesque CS Bideshi DK Jasinskiene N Coates CJ James AA Gene vector and transposable element behavior in mosquitoes J Exp Biol 2003 10 3823 3834 10.1242/jeb.00638 14506218

[B23] Peloquin JJ Thibault ST Staten R Miller TA Germ-line transformation of pink bollworm (Lepidoptera: gelechiidae) mediated by the piggyBac transposable element Insect Mol Biol 2000 10 323 333 10.1046/j.1365-2583.2000.00194.x 10886417

[B24] Williamson C Morris L Maughan MF Ping LH Dryga SA Thomas R Reap EA Cilliers T van HJ Pascual A Characterization and selection of HIV-1 subtype C isolates for use in vaccine development AIDS Res Hum Retroviruses 2003 10 133 144 10.1089/088922203762688649 12639249

[B25] van Harmelen JH Shephard E Thomas R Hanke T Williamson AL Williamson C Construction and characterisation of a candidate HIV-1 subtype C DNA vaccine for South Africa Vaccine 2003 10 4380 4389 10.1016/S0264-410X(03)00406-7 14505921

[B26] Ludwig C Wagner R Virus-like particles-universal molecular toolboxes Curr Opin Biotechnol 2007 10 537 545 10.1016/j.copbio.2007.10.013 18083549 PMC7126091

[B27] Halsey RJ Tanzer FL Meyers A Pillay S Lynch A Shephard E Williamson AL Rybicki EP Chimaeric HIV-1 subtype C Gag molecules with large in-frame C-terminal polypeptide fusions form virus-like particles Virus Res 2008 10 259 268 10.1016/j.virusres.2008.01.012 18329748

[B28] O'Reilly DR Miller LK Luckow VA Baculovirus Expression Vectors, A Laboratory Manual 1994 Oxford University Press. Inc

[B29] Mohammed A Coates CJ Promoter and piggyBac activities within embryos of the potato tuber moth, Phthorimaea operculella, Zeller (Lepidoptera: Gelechiidae) Gene 2004 10 293 301 10.1016/j.gene.2004.08.008 15527988

[B30] Gray CE Coates CJ High-level gene expression in Aedes albopictus cells using a baculovirus Hr3 enhancer and IE1 transactivator BMC Mol Biol 2004 10 8 10.1186/1471-2199-5-8 15251037 PMC487899

[B31] Huynh CQ Zieler H Construction of modular and versatile plasmid vectors for the high-level expression of single or multiple genes in insects and insect cell lines J Mol Biol 1999 10 13 20 10.1006/jmbi.1999.2674 10329122

[B32] Zieler H Huynh CQ Intron-dependent stimulation of marker gene expression in cultured insect cells Insect Mol Biol 2002 10 87 95 10.1046/j.0962-1075.2001.00312.x 11841506

[B33] Shi X Harrison RL Hollister JR Mohammed A Fraser MJ Jr Jarvis DL Construction and characterization of new piggyBac vectors for constitutive or inducible expression of heterologous gene pairs and the identification of a previously unrecognized activator sequence in piggyBac BMC Biotechnol 2007 10 5 10.1186/1472-6750-7-5 17233894 PMC1783651

[B34] Handler AM Harrell RA Germline transformation of Drosophila melanogaster with the piggyBac transposon vector Insect Mol Biol 1999 10 449 457 10.1046/j.1365-2583.1999.00139.x 10634970

[B35] Burgers WA van Harmelen JH Shephard E Adams C Mgwebi T Bourn W Hanke T Williamson AL Williamson C Design and preclinical evaluation of a multigene human immunodeficiency virus type 1 subtype C DNA vaccine for clinical trial J Gen Virol 2006 10 399 410 10.1099/vir.0.81379-0 16432028

[B36] Adelman ZN Jasinskiene N Vally KJ Peek C Travanty EA Olson KE Brown SE Stephens JL Knudson DL Coates CJ Formation and loss of large, unstable tandem arrays of the piggyBac transposable element in the yellow fever mosquito, Aedes aegypti Transgenic Res 2004 10 411 425 10.1007/s11248-004-6067-2 15587266

[B37] Wagner R Deml L Teeuwsen V Heeney J Yiming S Wolf H A recombinant HIV-1 virus-like particle vaccine: from concepts to a field study Antibiot Chemother 1996 10 68 83 8726508 10.1159/000425160

[B38] Jarvis DL Weinkauf C Guarino LA Immediate-early baculovirus vectors for foreign gene expression in transformed or infected insect cells Protein Expr Purif 1996 10 191 203 10.1006/prep.1996.0092 8812860

[B39] Leisy DJ Rasmussen C Owusu EO Rohrmann GF A mechanism for negative gene regulation in Autographa californica multinucleocapsid nuclear polyhedrosis virus J Virol 1997 10 5088 5094 9188574 10.1128/jvi.71.7.5088-5094.1997 PMC191742

[B40] Maragathavally KJ Kaminski JM Coates CJ Chimeric Mos1 and piggyBac transposases result in site-directed integration FASEB J 2006 10 1880 1882 10.1096/fj.05-5485fje 16877528

[B41] Iizuka M Tomita M Shimizu K Kikuchi Y Yoshizato K Translational enhancement of recombinant protein synthesis in transgenic silkworms by a 5'-untranslated region of polyhedrin gene of Bombyx mori Nucleopolyhedrovirus J Biosci Bioeng 2008 10 595 603 10.1263/jbb.105.595 18640598

[B42] Imamura M Nakai J Inoue S Quan GX Kanda T Tamura T Targeted gene expression using the GAL4/UAS system in the silkworm Bombyx mori Genetics 2003 10 1329 1340 14668386 10.1093/genetics/165.3.1329 PMC1462818

[B43] Horn C Handler AM Site-specific genomic targeting in Drosophila Proc Natl Acad Sci USA 2005 10 12483 12488 10.1073/pnas.0504305102 16116081 PMC1194931

[B44] Sarkar A Atapattu A Belikoff EJ Heinrich JC Li X Horn C Wimmer EA Scott MJ Insulated piggyBac vectors for insect transgenesis BMC Biotechnol 2006 10 27 10.1186/1472-6750-6-27 16776846 PMC1525164

[B45] Liu B Paton JF Kasparov S Viral vectors based on bidirectional cell-specific mammalian promoters and transcriptional amplification strategy for use in vitro and in vivo BMC Biotechnol 2008 10 49 10.1186/1472-6750-8-49 18485188 PMC2396617

[B46] Tsunetsugu-Yokota Y Morikawa Y Isogai M Kawana-Tachikawa A Odawara T Nakamura T Grassi F Autran B Iwamoto A Yeast-derived human immunodeficiency virus type 1 p55(gag) virus-like particles activate dendritic cells (DCs) and induce perforin expression in Gag-specific CD8(+) T cells by cross-presentation of DCs J Virol 2003 10 10250 10259 10.1128/JVI.77.19.10250-10259.2003 12970409 PMC228384

[B47] Hu YC Baculovirus as a highly efficient expression vector in insect and mammalian cells Acta Pharmacol Sin 2005 10 405 416 10.1111/j.1745-7254.2005.00078.x 15780188 PMC7091893

[B48] Sinn PL Burnight ER Hickey MA Blissard GW McCray PB Jr Persistent gene expression in mouse nasal epithelia following feline immunodeficiency virus-based vector gene transfer J Virol 2005 10 12818 12827 10.1128/JVI.79.20.12818-12827.2005 16188984 PMC1235842

[B49] Abe T Takahashi H Hamazaki H Miyano-Kurosaki N Matsuura Y Takaku H Baculovirus induces an innate immune response and confers protection from lethal influenza virus infection in mice J Immunol 2003 10 1133 1139 12874198 10.4049/jimmunol.171.3.1133

[B50] Li Z Blissard GW Functional analysis of the transmembrane (TM) domain of the Autographa californica multicapsid nucleopolyhedrovirus GP64 protein: substitution of heterologous TM domains J Virol 2008 10 3329 3341 10.1128/JVI.02104-07 18216100 PMC2268458

[B51] Hervas-Stubbs S Rueda P Lopez L Leclerc C Insect baculoviruses strongly potentiate adaptive immune responses by inducing type I IFN J Immunol 2007 10 2361 2369 17277142 10.4049/jimmunol.178.4.2361

[B52] Zhao H Janke M Fournier P Schirrmacher V Recombinant Newcastle disease virus expressing human interleukin-2 serves as a potential candidate for tumor therapy Virus Res 2008 10 75 80 10.1016/j.virusres.2008.04.020 18538434

[B53] Dafa'alla TH Condon GC Condon KC Phillips CE Morrison NI Jin L Epton MJ Fu G Alphey L Transposon-free insertions for insect genetic engineering Nat Biotechnol 2006 10 820 821 10.1038/nbt1221 16823373

